# Potential and Challenges of Transcranial Photobiomodulation for the Treatment of Stroke

**DOI:** 10.1111/cns.70142

**Published:** 2024-12-18

**Authors:** Siyue Li, Thomson W. L. Wong, Shamay S. M. Ng

**Affiliations:** ^1^ Department of Rehabilitation Sciences The Hong Kong Polytechnic University Hong Kong SAR China

**Keywords:** animal studies, clinical trials, low‐level laser therapy, photobiomodulation, stroke

## Abstract

Photobiomodulation (PBM), also known as low‐level laser therapy, employs red or near‐infrared light emitted from a laser or light‐emitting diode for the treatment of various conditions. Transcranial PBM (tPBM) is a form of PBM that is delivered to the head to improve brain health, as tPBM enhances mitochondrial function, improves antioxidant responses, reduces inflammation, offers protection from apoptosis, improves blood flow, increases cellular energy production, and promotes neurogenesis and neuroplasticity. As such, tPBM holds promise as a treatment for stroke. This review summarizes recent findings on tPBM as a treatment for stroke, presenting evidence from both animal studies and clinical trials that demonstrate its efficacy. Additionally, it discusses the potential and challenges encountered in the translation process. Furthermore, it proposes new technologies and directions for the development of light‐delivery methods and emphasizes the need for extensive studies to validate and widen the application of tPBM in future treatments for stroke.

## Graphical abstract

tPBM employs red or near‐infrared light to enhance mitochondrial function, promote neurogenesis, and reduce neuroinflammation, offering potential benefits for stroke recovery. This review summarizes evidence from animal studies and clinical trials validating tPBM's efficacy and discusses translational challenges and future directions in treatment advancement.

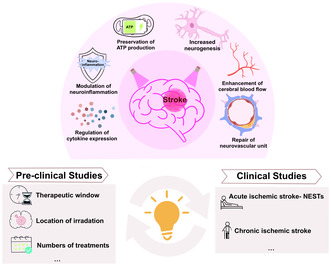

## 1 Introduction

Photobiomodulation (PBM), formerly known as low‐level laser therapy and then low‐level light therapy, is a promising modality that involves the irradiation of cells and tissues with photons of specific wavelength ranges. This non‐invasive and non‐thermal approach stimulates a biological response, particularly at red and near‐infrared (NIR) wavelengths (600–700 nm and 760–1200 nm, respectively) and extending to blue and green wavelengths [1, 2, 3]. The availability of cost‐effective and safe light‐emitting diodes (LEDs) rendered the use of expensive lasers unnecessary in many cases, leading to low‐level laser therapy being re‐named as low‐level light therapy (LLLT). Subsequently, to solve the problem of “low level” being ambiguous, LLLT was renamed as PBM [4, 5]. NIR light has gained popularity in PBM due to its tissue penetration properties and overall efficacy being superior to those of other wavelengths of light [6, 7, 8]. PBM has been applied in many treatments, such as for wound healing stimulation [9], pain reduction [10], inflammation alleviation in musculoskeletal conditions [11, 12, 13], and mitigation of cancer‐therapy side effects [14, 15]. Moreover, in recent years, there has been a growing interest in exploring the use of transcranial PBM (tPBM) for treating various neurological diseases, such as stroke [16, 17, 18], traumatic brain injury (TBI) [19, 20], Alzheimer's disease (AD) [21, 22], Parkinson's disease (PD) [1, 23], and depression [4].

tPBM is considered to be a safe intervention with no deleterious effects on the structure and function of the brain [24, 25]. However, the inconsistencies in efficacy observed across various studies have raised questions about its optimal application and the underlying mechanisms that govern its effects. Significant challenges, such as the determination of effective dosimetry parameters, and variation in treatment protocols, must be addressed to fully realize its potential for clinical use. In this article, we review recent findings on the potential of tPBM for stroke and summarize lessons learned from previous animal and human studies. We aim to identify potentials and challenges that may be encountered during the translation of tPBM from the laboratory to the clinic for use as a cerebral protective or restorative tool in stroke management. As such, we hope that this review will enhance the understanding and application of tPBM in the treatment of stroke.

## 2 Roles of tPBM for Stroke Recovery

**FIGURE 1:**
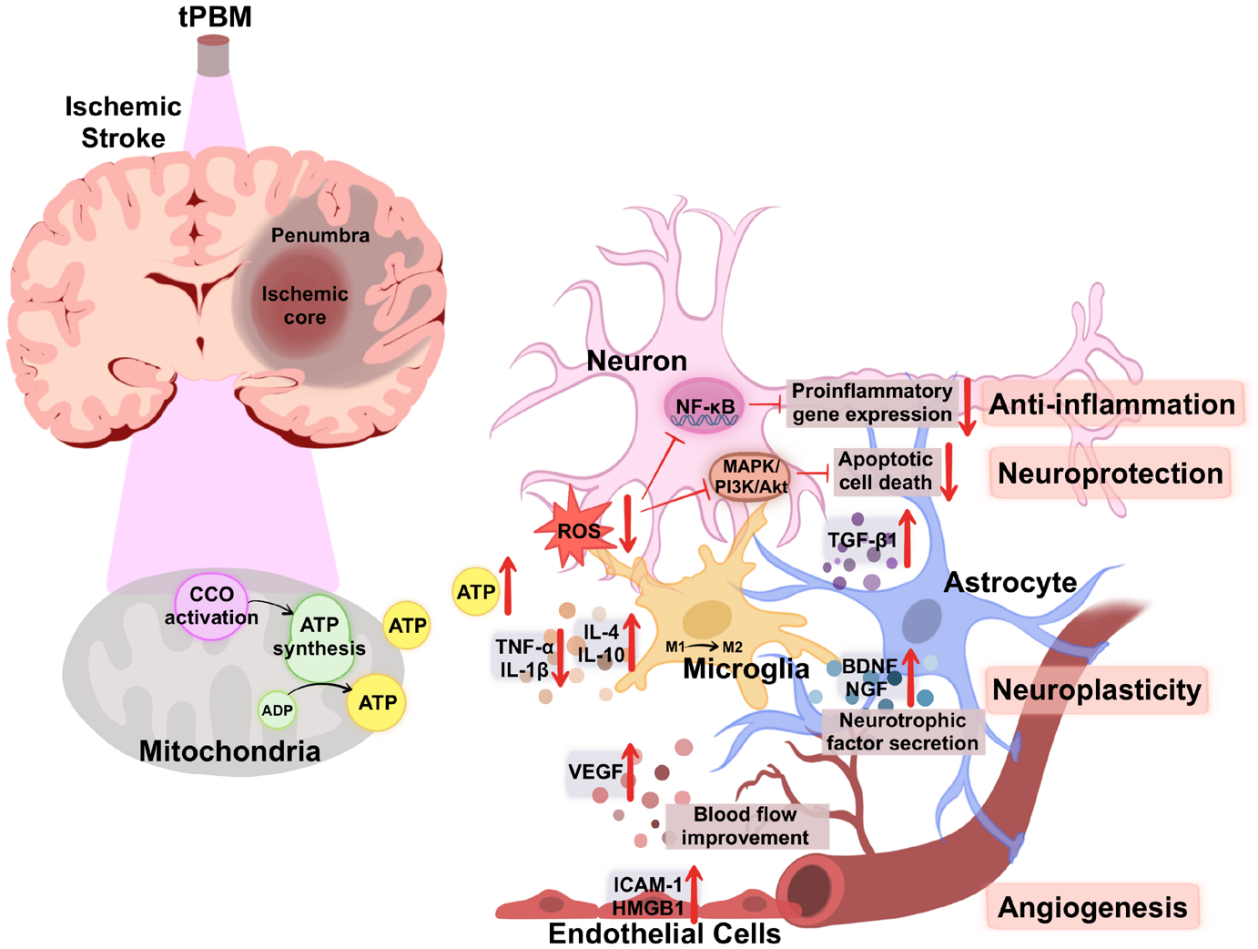
Schematic representation of tPBM effects in ischemic stroke. tPBM delivers red and NIR light, which is absorbed by the mitochondrial chromophore‐CCO. This absorption promotes ATP generation and affects ROS production, activates transcription factors and signaling pathways. These cascades lead to various photobiological effects, including reduced oxidative stress, neuronal apoptosis, inflammation, and increased neurotrophic levels, angiogenesis, and neuroplasticity, contributing to brain recovery in ischemic stroke.

Stroke is a complex and dynamic process characterized by both acute and reparative phases. In the early acute phase of ischemic stroke, the disruption of oxygen and glucose supply to the brain leads to mitochondrial dysfunction, which is a hallmark of hypoxic‐ischemic brain injury [26]. tPBM treatment applied in this phase targets neurons by utilizing wavelengths of light that can be absorbed by chromophores, such as cytochrome C oxidase (CCO), the primary photoreceptor for red‐to‐NIR light in the mitochondria of neurons. The stimulation of CCO leads to an increase in ATP production, a decrease in mitochondrial nitric oxide (NO) concentrations, and a reduction in reactive oxygen species (ROS) concentrations [27], all of which alleviate oxidative stress and inflammation, thereby helping to mitigate neuronal damage [28]. As stroke progresses to subacute and chronic phases, tPBM treatment enhances hemodynamic and neuroplasticity to provide bioenergetic relief and also enhances various biological reactions. tPBM treatment activates secondary cell‐signaling pathways, enhances cerebrovascular oxygenation, increases cerebral blood flow (CBF), exerts anti‐inflammatory effects, and promotes the production of endogenous neurotrophic factors, such as nerve growth factor and brain‐derived neurotrophic factor (BDNF) [25]. These molecular effects of tPBM treatment contribute to the remodeling of synaptogenesis, neurogenesis, and neuroplasticity, resulting in long‐lasting support of stroke recovery. In addition, tPBM has been shown to generate considerable improvements at the behavioral level after stroke, such as improvements in motor skills, memory, mood, and sleep quality [29, 30, 31]. In summary, tPBM treatment is able to activate endogenous mechanisms to promote neuroprotection and repair damaged neuronal pathways throughout stroke recovery (Figure 1).

Furthermore, the beneficial effects of tPBM may not solely rely on direct light penetration into brain cells, as it can also have systemic effects. That is, tPBM irradiates the blood, enhancing the activity of circulating blood, resulting in positive effects beyond the irradiated region, a phenomenon known as “remote photobiomodulation” [32, 33]. For example, tPBM treatment of patients with PD led to significant mobility improvements [34], despite it being unlikely that tPBM directly protects vulnerable brain cells located in deep brain regions such as the nigrostriatal pathway. Instead, these benefits may stem from enhanced neural function and communication throughout the brain. Similarly, studies have demonstrated that tPBM had beneficial effects on brain histology even when the head was shielded from direct light [35], highlighting the remote effects of tPBM applied to areas other than the head [36, 37].

## 3 tPBM Therapy in Animal Models

Preclinical testing of tPBM therapy in animal models has shown promising results in decreasing infarct size, improving motor function, reducing long‐term neurological deficits and mortality, and enhancing spatial learning and memory (Table 1) [38, 39, 40, 41, 42, 43]. However, there may be variability in the experimental protocols used, and various methodological factors can significantly influence the outcomes of both preclinical and clinical stroke treatment studies.

**TABLE 1:** Summary of t‐PBM studies on stroke in animal models.

Authors	Animal model	Parameters of light	Onset of stroke	Irradiation sites	Duration time	Evaluation tools	Outcomes
Leung 2002 [44]	SD rat, 1 h transient MCAO	Laser, 660 nm, average power 8.8 mW, energy density 2.64 J per cm^2^ /min, pulse frequency 10 kHz	Immediately after stroke	Cerebrum	1, 5, or 10 min	NOS, TGF‐β1	Low energy laser suppressed NOS activity and up‐regulated TGF‐β1 expression
Lapchak 2004 [45]	Rabbit, RSCEM	Laser, 808 ± 5 nm, 7.5 or 25 mW/cm^2^, CW	1, 3, 6, or 24 h after embolization	Posterior to bregma on the midline, both ipsilateral and contralateral sides	2 or 10 min	Effective stroke dose (P_50_), clot amount (mg)	Laser treatment improved behavioral performance if initiated within 6 h and the effect was durable
DeTaboada 2006 [46]	SD rat, permanent MCAO	Laser, 808 nm, 7.5 mW/cm^2^, CW	24 h post‐stroke	3 mm dorsal to the eye and 2 mm anterior to the ear, ipsilateral, contralateral, and bilateral	2 min at each point	Modified neurological score	LLLT improved neurological deficits at delayed 14, 21, and 28 days post‐stroke at different skull locations
Oron 2006 [47]	(1) SD rat; (2) Wistar rats, permanent MCAO	Laser, 808 nm, 7.5 mW/cm^2^, (1) CW; (2) both CW and PW (70 Hz)	4 and 24 h post‐stroke	3 mm dorsal to the eye and 2 mm anterior to the ear, contralateral	2 min at each point	Modified neurological severity scores, infarct volume, BrdU, DCX	LLLT issued 24 h after acute stroke induced functional benefit and neurogenesis
Lapchak 2007 [48]	Rabbit, RSCEM	Laser, 808 ± 5 nm, power density 7.5 mW/cm, (1) CW; (2) PW, 300 μs pulse at 1 kHz; (3) PW, 2 ms pulse at 100 Hz	6 or 12 h following embolization	Posterior to bregma on the midline	2 min	Effective stroke dose (P_50_), clot amount (mg)	PW mode NILT resulted in significant clinical improvement within 6 h from stroke onset
Lapchak 2008 [49]	Rabbits, large clot embolism‐induced stroke	Laser, 808 ± 5 nm, power density 10 mW/cm^2^	90 min after embolization	Posterior to bregma on the midline	2 min	Hemorrhage rate, volume	TLT administration did not affect the tPA‐induced increase in hemorrhage incidence. TLT may be administered safely either alone or in combination with tPA
Lapchak 2010 [50]	Rabbit, RSCEM	Laser, 808 nm, (1) 7.5 mW/cm^2^, cortical fluence 0.9 J/cm^2^, CW; (2) 37.5 mW/cm^2^, cortical fluence 4.5 J/cm^2^, PW, 100 Hz; (3) 262.5 mW/cm^2^, cortical fluence 31.5 J/cm^2^, PW, 100 Hz	5 min post‐embolization	Posterior to bregma on the midline	2 min	ATP	NILT increased cortical ATP content, and this was correlated with cortical fluence and the mode of NILT delivery. PW NILT delivered 5 and 35 times more energy than CW
Uozumi 2010 [51]	C57BL/6J mice, transient BCCAO	Laser, 808 nm, power densities 0.8, 1.6 and 3.2 W/cm^2^	30 min before BCCAO	Left hemisphere, 2 mm posterior to and 3 mm left of the bregma	15–45 min	CBF, brain temperature, TUNEL	NIR laser irradiation increased cerebral blood flow and was concerned with NOS activity and NO concentration
Yip 2011 [52]	SD rat, 1 h transient MCAO	Laser, 660 nm, average power 8.8 mW, 10 kHz, 2.64 J/cm^2^, 13.2 J/cm^2^, and 24.6 J/cm^2^	Immediately following MCAO	Cerebrum	1, 5, or 10 min	Akt, BAD, Bcl‐2, caspase 9, caspase 3	LLI protected the brain by upregulating Akt, pAkt, pBAD, and Bcl‐2 expression and downregulating caspase 9 and caspase 3 expression
Huisa 2013 [53]	Rabbit, RSCEM	Laser, 808.5 nm, 7.5–20 mW/cm^2^, 10.8 mW/cm^2^ for the single therapy and 20 mW/cm^2^ for the triple therapy	2, 3, 4 and 5 h post‐embolization	Skin overlying the skull	2 min for each dose	Effective stroke dose (P_50_), clot amount (mg)	Triple treatment had a greater improvement when compared with single or sham treatment
Lapchak 2016 [54]	Rabbit, RSCEM	Laser, 808 ± 5 nm, 7.5 mW/cm^2^, CW	1 h post embolization	Posterior to bregma on the midline	2 min	Effective stroke dose (P_50_), ICH rate, ATP	Combination of TLT‐tPA enhanced ATP production, and produced an additive effect on ATP levels
Lee 2016 [55]	C57BL/6J mice, PT	LED, 610 nm, power intensity 1.7 mW/cm^2^, energy density 2.0 J/cm^2^	2 days before the ischemic event	Right midpoint of the parietal bone and the posterior midline of the seventh cervical vertebra	20 min, twice a day for 2 days	Infarct volume, COX‐2, p65, p38, JNK, ERK, iNOS, TNF‐α, IL‐1β, IL‐6	LLLT improved functional benefits by suppressing neuroinflammation, such as inhibiting inflammatory mediators and MAPK/NF‐κB activation. LLLT also prevented BBB disruption
Meyer 2016 [56]	Rabbit, RSCEM	Laser, 808.5 nm, 111 mW, 100 Hz	2 h post‐embolization	Head	2 min	ES50, the weight of clots (mg)	TLT improved behavior with a triple TLT regimen
Lee 2017 [39]	C57BL/6J mice, PT	LED, 610 nm, 1.7 mW/cm^2^, 2.0 J/cm^2^	4 h post‐ischemia	Right midpoint of the parietal bone and the posterior midline of the seventh cervical vertebra	20 min, twice a day for 3 days	Infarct volume, neurological score, NF‐κB p65, IL‐1β, IL‐18, JNK, ERK, NLRP3, TUNEL	LED therapy reduced infarct, improved function, and decreased neuroinflammation in the ischemic cortex. It attenuated the NLRP3 inflammasome, suppressed TLR‐2/MAPK/NF‐κB, and decreased cell death
Lee 2017 [38]	C57BL/6J mice, PT	LED, 610 nm, power intensity 1.7 mW/cm^2^, energy density 2.0 J/cm^2^	Immediately, 4 days or 10 days after ischemia	Right midpoint of the parietal bone and the posterior midline of the seventh cervical vertebra	20 min, once a day for 7 days	Behavior tests, brain atrophy, BrdU, Iba‐1, NeuN, DCX, CD31, BNDF	Acute and subacute LED therapy improves long‐term functional recovery through neuron/astrocyte proliferation, angiogenesis, and increased BDNF
Lee 2017 [57]	C57BL/6J wild‐type and eNOS‐deficient mice, MCAO	LED, 610 nm, power intensity 1.7 mW/cm^2^, energy density 2.0 J/cm^2^	Before cerebral ischemia	Right midpoint of the parietal bone and the posterior midline of the seventh cervical vertebra	Twice a day for 20 min for 2 days	Brain infarct, edema volume, neurological scales, eNOS, Akt	Pretreatment with LED reduced brain damage by the stimulation of eNOS phosphorylation via the PI3K/Akt pathway
Sanderson 2018 [58]	SD, global brain ischemia and reperfusion	LED, 750 nm, 810 nm, 950 nm, power density at the scalp 50 mW/cm^2^	At the onset of reperfusion	Intact scalp and skull	120 min	COX, ROS, mitoSOX, Iba‐1, NeuN	NIR therapy preserved neurologic function, and protected neuron loss in CA1 hippocampus post‐reperfusion
Yang 2018 [59]	Rats, PT	Laser, 808 nm, 25 mW/cm^2^ at cerebral cortex tissue level, 350 mW/cm^2^ on the scalp, CW	1 day following PT	Infarct injury area (1.8 mm anterior to the bregma and 2.5 mm lateral from the midline)	2 min daily, from day 1 to day 7	Infarct volume, BrdU, NeuN, MAP2, DCX, Iba‐1, CCO, ATP, IL‐4, IL‐10, IL‐6, TNF‐α, IL‐18, CD32, CD86, iNOS, TGF‐β, CD206	PBM promoted neurogenesis after ischemic stroke. The mechanisms may rely on (1) promotion of proliferation and differentiation of internal neuroprogenitor cells in the peri‐infarct zone; (2) improvement of the neuronal microenvironment by altering inflammatory status and promoting mitochondrial function
Argibay 2019 [60]	SD rat, 1 h transient MCAO	LED, 830 nm, 10 mW/cm^2^, 0.28 J/cm^2^ at brain cortex, CW	24 h after stroke	Head	(1) 30 min, 1 day/week for 12 weeks; (2) 30 min, 3 days/week for 12 weeks	Infarct volume, sensorimotor test, Fox3, Ki‐67, DCX	PBM did not reduce infarct size or improve functional recovery
Fonseca 2019 [61]	Wistar rat, hemiplegia, electrode implanted in the internal capsule	LED, 904 nm, 110 mW, 7 J/cm^2^	Day 4, 7, 21 post‐stroke	Frontal region of the brain	63 s, daily, for 3, 7, and 21 days	Neurological behavior test, H&E staining	LED irradiation may beneficially affect neurogenesis, reduce edema and density of the cerebral parenchyma, increase muscle resistance and animal motor behavior, especially on treatment days 7 and 21
Salehpour 2019 [62]	BALB/c mice, 20 min transient BCCAO	Laser, 810 nm, irradiance 6.66 W/cm^2^, 200 mW maximum output power	On the day of ischemia	Midline of the dorsal surface of the head in region between eyes and ears	Once a day for 14 days	Neurological severity score, ROS, ATP, SIRT1, PGC1‐α, iNOS, TNF‐α, IL‐1β	PBM and Coenzyme Q10, alone or combined, had beneficial effects by reducing neuroinflammatory markers like iNOS, TNF‐α, and IL‐1β
Vahabzadeh‐Hagh 2019 [63]	C57BL/6J mice, mouse focal cerebral ischemia model	Laser, 808 nm, 37 mW/cm^2^, 4.4 J/cm^2^, CW	At day 5, 9, and 13 after stroke onset	Head	2 min	MAP2, HMGB1, CD38, BDNF, IGF‐1, FGF‐2, VEGF, CD34	Repeated NIR irradiation increased HMGB1 in the peri‐infarct cortex, leading to higher accumulation of EPCs
Strubakos 2020 [64]	Rat, 90 min transient right MCAO	LED, 750 and 950 nm, 200 mW/cm^2^	Immediately initiated at reperfusion onset	1.5 cm from the shaved scalp	120 or 240 min	Infarct volume	NIR attenuated brain injury and evoked a sustained reduction in infarct volume following ischemic stroke
Guo 2021 [65]	Rats, four‐vessel occlusion, GCI	Laser, 808 nm, irradiance 20 mW/cm^2^, fluence at cortical surface 2.4 J/cm^2^ and at the hippocampus 0.8 J/cm^2^	3 days after GCI	3 mm posterior to the eye and 2 mm anterior to the ear	2 min, for 5 days	BrdU, DCX, NeuN, NLRP3, IL‐1β, Iba‐1, ultrastructure of astrocyte and microglia	PBM had long‐term protective effects on astrocytes and promoted hippocampal neurogenesis, contributing to neurological recovery
Vogel 2021 [41]	Wistar rat, PT	Laser, 780 nm, 10 mW/cm^2^, intensity of 0.083 W/cm^2^, energy density of 10 J/cm^2^	24 h after PT	Injury area (positioned 1 mm posterior and 1 mm lateral to bregma)	2 min, alternate days, 3 times/week, for 60 days	Ischemia volume, Iba, NeuN, TNF‐α, IL‐1β, IL‐6, IL‐10, TGF‐β	PBM reduced lesion volume, microglial activation, and neuroinflammation, while increasing astroglial activity in the peri‐lesioned region
Vogel 2021 [43]	Wistar rats, PT	Laser, 780 nm, 10 mW, 0.083 W/cm^2^, 10 J/cm^2^	24 h after PT	Injured area (1 mm posterior and 1 mm lateral from bregma)	2 min, 3 times/week, for 60 days	Electroencephalogram	PBM reduced epileptiform discharges in stroke‐induced epilepsy
Kim 2022 [66]	C57BL/6 mice, PT and MCAO	LED, 630 nm, 850 nm, 940 nm, 17 mW/ cm^2^	Before the induction of ischemia as well as 1 h before the procedure	Sensorimotor cortex of the ipsilesional region	(1) 20 min, twice a day before or after PT for 3 days; (2) immediately after PT, once daily for 7 days	Infarct volume, neurological scores, NeuN, CD31, Iba‐1, AIM2, caspase 1, GS‐ DMD, CD86, CD206, IL‐1β, IL‐10	PBM attenuated inflammasome activation, inflammasome‐mediated pyroptosis, and modulated microglial polarization in the hippocampus and cortex 7 days post‐stroke
Feng 2023 [67]	SD rat, PT	Laser, 808 nm, power density 350 mW/cm^2^ at the scalp level	Initiated 24 h after PT	1.8 mm anterior to the bregma and 2.5 mm lateral from the midline	2 min daily, for 7 days	Synaptophysin, IL‐1β, Bcl‐xL, BAX, Spinophilin, NeuN, MAP2, Caspase 9, C3d, iNOS	PBM inhibited neurotoxic astrocytic polarization, preserved synaptic integrity and protected neurons against stroke injury both in vitro and in vivo
Shalaby 2023 [68]	C57BL/6 mice, PT in the olfactory bulb	Laser, 808 nm, 325 mW/cm^2^, fluence of 40 J/cm^2^	From day 2	The olfactory bulb area	2 min daily, from day 2 to day 7	Iba‐1, CD31	PBM improved olfactory recovery after PT by modulating the micro‐environment, suppressing inflammatory cytokines and enhancing glial and vascular factors
Feng 2024 [69]	SD rats, PT	Laser, 808 nm, 350 mW/cm^2^, 42 J/cm^2^	From day 1	1.8 mm anterior to the bregma and 2.5 mm lateral from the midline.	2 min daily, from day 1 to day 7	CBF, blood–brain barrier permeability, ZO‐1, Claudin 5, testosterone	PBM attenuated cerebrovascular injury and behavioral deficits associated with testosterone/androgen receptor following ischemic stroke
Yokomizo 2024 [70]	C57BL/6J, 30 min MCAO	Laser, 808 nm, 1064 nm, 1270 nm, irradiance 50 mW/cm^2^, CW	Pretreatment	Head	5 min daily for 4 days before stroke	CBF, infarct volume, eNOS	Laser pretreatment laser improved cerebral blood flow, eNOS phosphorylation, and stroke outcomes

Abbreviations: AIM, interferon‐inducible protein; BBB, blood–brain barrier; BCCAO, bilateral common carotid artery occlusion; BNDF, brain‐derived neurotrophic factor; BrdU, bromodeoxyuridine; CA1, hippocampal cornu ammonis; CBF, cerebral blood flow; CCO, cytochrome c oxidase; CD, cluster of differentiation; COX, cyclooxygenase; CW, continuous wave; DCX, doublecortin; eNOS, endothelial nitric oxide synthase; EPC, endothelial progenitor cell; ERK, extracellular signal‐regulated kinase; FGF, fibroblast growth factor; GCI, global cerebral ischemia; HMGB, high‐mobility group box 1; Iba, ionized calcium‐binding adapter molecule 1; ICH, intracerebral hemorrhage; IGF, insulin like growth factor; IL, interleukin; iNOS, inducible nitric oxide synthase; JNK, c‐Jun N‐terminal kinase; LED, light‐emitting diodes; LLLT, low‐level light therapy; MAP, microtubule‐associated protein; MAPK, mitogen‐activated protein kinase; MCAO, middle cerebral artery occlusion; NF‐κB, nuclear factor kappa‐light‐chain‐enhancer of activated B cells; NILT, near‐infrared light therapy; NIR, near‐infrared; NLRP3, NLR family pyrin domain containing 3; NOS, nitric oxide synthase; PGC1‐α, peroxisome proliferator‐activated receptor gamma coactivator; PT, photo thrombosis; PW, pulse wave; ROS, reactive oxygen species; RSCEM, rabbit small clot embolic stroke model; SIRT, NAD‐dependent deacetylase sirtuin; TGF, transforming growth factor; TLR, toll‐like receptor; TLT, transcranial laser therapy; TNF, tumor necrosis factor; tPA, tissue‐type plasminogen; TUNEL, terminal deoxynucleotidyl transferase dUTP nick end labeling; VEGF, vascular endothelial growth factor.

### 3.1 Therapeutic Window

The timing of tPBM treatment initiation post‐stroke has been investigated in animal studies. In the rabbit embolic stroke model (RSCEM), initiation of laser treatment within 3–6 h significantly improved behavioral performance for up to 3 weeks, but initiation at 24 h did not [45]. However, in the rat permanent middle cerebral artery occlusion (MCAO) model, laser treatment at 24 h resulted in neurological function improvement at 3–4 weeks [46, 47]. In addition, LED treatment during the subacute phase (4 days post‐stroke onset) significantly improved motor function up to 4 weeks, but a later phase at 10 days post‐stroke onset did not [38, 61]. Studies have also examined combining tPBM with tissue‐type plasminogen activator (tPA) therapy [49, 54]. The therapeutic window of tPA is 1–1.5 h in the RSCEM, while LLLT had a 4–6 times longer window. Importantly, LLLT did not increase hemorrhagic incidence, volume or mortality rate. In turn, there was a 30% decrease in hemorrhagic incidence when tPA was combined with tPBM [49]. Above evidence suggests that the therapeutic window for tPBM varies by animal model and stroke induction method, but can be extended as a synergistic approach with tPA. It can also exert long‐term protective effects and modulate brain responses at different stages of the ischemic cascade.

### 3.2 Location of Irradiation

Studies have suggested that both focal and whole‐brain approaches to tPBM may have advantages for promoting neurological recovery after stroke. Irradiating a stroked hemisphere ipsilaterally, contralaterally, or bilaterally improved neurological outcomes in rats, with effects observed 24 h after stroke induction and persisting for 4 weeks [46]. Notably, evidence suggests that whole‐brain treatment may be more effective than focusing solely on the ipsilateral side, as beneficial effects have also been observed with contralateral irradiation [46]. Though the optimal side for irradiation in animal studies remains unclear, targeting the entire brain may generally yield more favorable results. This has prompted subsequent clinical trials to favor whole‐brain irradiation [17, 71], while later studies have explored the efficacy of targeting specific brain regions [18, 72].

### 3.3 Number of Treatments

The numbers and total energy dose of tPBM treatments may optimize the therapeutic response. Double or triple laser treatments with a surface power density of 7.5–20 mW/cm^2^ within 5 h post‐embolization resulted in greater behavioral improvements than a single treatment [53]. Specifically, the triple treatment group showed a remarkable 245% behavioral improvement over controls, exceeding that of neuroprotective agents like tPA [53]. Similarly, triple high‐power treatments also provided significant behavioral benefits without causing tissue damage [56].

### 3.4 Mechanisms of Effects of tPBM on the Brain in Animal Models

#### 3.4.1 Preservation of ATP Production and Protection of Mitochondria

tPBM treatment has been shown to increase ATP concentrations and rescue mitochondrial dysfunction in neurons in ischemic animal stroke models [50, 59], highlighting its potential for stroke treatment. NIR laser irradiation was observed to suppress nitric oxide synthase (NOS) activity and expression, alleviating cerebral concentrations of NO in transient MCAO rat models [44, 51, 52]. Additionally, LED preconditioning stimulates endothelial NOS phosphorylation via the phosphoinositide 3‐kinase/Akt pathway, leading to reductions in brain damage in mice after MCAO [57, 70]. Moreover, tPBM treatment targeting mitochondria attenuated ischemia–reperfusion injury by addressing the initial ROS burst, thereby reducing brain injury and infarct volume in the rat MCAO model [64]. tPBM was also observed to protect primary cortical neurons against excitotoxicity induced by glutamate or kainite [67], which are major contributors to neuronal death in ischemic stroke. Furthermore, tPBM has been found to indirectly protect tissues by inhibiting apoptosis. It downregulates pro‐apoptotic factors like caspase 9 and caspase 3, and upregulates anti‐apoptotic factors like Akt, Bcl‐2 [52]. tPBM treatment has also been shown to improve muscle resistance, physical function, and spatial and episodic memory, and increase markers of mitochondrial biogenesis [61, 62].

#### 3.4.2 Increased Neurogenesis

tPBM has been found to promote cortical neurogenesis by stimulating neural progenitor cells and neural stem cells in key neurogenic niches like the subventricular zone (SVZ) and dentate gyrus. Studies have shown that tPBM treatment initiated within 24 h of ischemia can significantly enhance neurogenesis [73]. For example, LED irradiation of the hemisphere contralateral to the stroked region resulted in an increase in newly formed neuronal cells in the ipsilateral SVZ [47]. Similarly, tPBM treatment initiated up to 6 h after an embolic stroke could induce rapid behavioral improvements, along with increased number of nerve‐producing cells in the angular gyrus, and elevated BDNF concentrations [48]. Moreover, daily LED irradiation was reported to improve the histological appearances of oligodendrocytes, pyramidal motor neurons, and sensory neurons, reflecting enhanced neurogenesis [61]. In vitro, LED irradiation of rat cortical neurons exposed to ischemic conditions promoted neurite outgrowth and synaptogenesis, mediated by the activation of mitogen‐activated protein kinase (MAPK) signaling [74]. However, the positive effects of tPBM treatment on post‐stroke neurogenesis may take 2–4 weeks to manifest, as new neurons are required to form and migrate to the damaged site. Studies have shown that tPBM treatment during acute and subacute phases of stroke can induce the proliferation of bromodeoxyuridine (BrdU)‐positive cells, as well as BrdU co‐labeling with markers of astrocytes, neuronal precursors, mature neurons, and blood vessels [38]. Relatedly, in a photothrombotic stroke model, daily tPBM treatment applied to an infarct area significantly increased BrdU immunostaining of proliferation, neuronal, dendritic spine, and synaptic markers in the peri‐infarct zone [59]. Moreover, in a global cerebral ischemia model, tPBM treatment suppressed the activity of reactive astrocytes and maintained astrocyte regeneration as early as 7 days post‐reperfusion, and promoted the formation of new neurons at 58 days post‐reperfusion [65].

#### 3.4.3 Modulation of Neuroinflammation and Regulation of Cytokine Expression

tPBM treatment has been found to downregulate pro‐inflammatory cytokines that can promote neuronal apoptosis, aggravate brain injury, inhibit neurogenesis, and disrupt the integrity of the blood–brain barrier. Conversely, tPBM has been shown to upregulate anti‐inflammatory cytokines, indicating its potential to mitigate neuroinflammation [25]. For example, tPBM treatment before photothrombotic cortical ischemia inhibited MAPK activation and NF‐κB translocation, prevented leukocyte accumulation, and preserved the integrity of the blood–brain barrier [55]. It also decreased expression of the NLRP3 inflammasome, which is responsible for cleaving pro‐inflammatory cytokines IL‐1β and IL‐18 [65, 75]. tPBM treatment initiated 4 h post‐ischemia profoundly reduced neuroinflammatory responses, including neutrophil infiltration and microglial activation, as well as decreases in the concentrations of inducible NOS, TNF‐α, and IL‐1β in the ischemic cortex [62]. Irradiation of the olfactory bulb with tPBM significantly decreased the expression of pro‐inflammatory cytokines, such as IL‐1α, IL‐1β, and IL‐16, while increasing the expression of anti‐inflammatory cytokines, such as IL‐1 receptor antagonist, IL‐4, and IL‐10 [68]. In addition, tPBM treatment was found to modulate microglial activation, shifting microglia from the pro‐inflammatory M1 phenotype to the anti‐inflammatory M2 phenotype [41], thereby promoting an environment conducive to regeneration and improved mitochondrial function and structure. Furthermore, tPBM treatment attenuated apoptosis and inflammasome activation in the hippocampus and cortex following ischemic stroke [66, 67], contributing to significant improvements in neurological behavior [74].

#### 3.4.4 Enhancement of CBF and Repair of the Neurovascular Unit

tPBM treatment has shown a potential to enhance local CBF by 30% in a mouse model [51], likely due to increases in NO and reductions in hippocampal apoptosis. tPBM treatment of the olfactory bulb not only accelerated the recovery of impaired olfaction by enhancing the expression of glial and vascular factors, such as GFAP, Iba‐1, and CD31 [68] but also increased the concentration of soluble intercellular adhesion molecule‐1, which is associated with cerebral microbleeds and hemorrhagic transformation in stroke. Studies also indicate that tPBM treatment can modulate intercellular signaling within the neurovascular unit. It triggers reactive astrocytes to release high‐mobility group box 1, which may enhance the accumulation of endothelial progenitor cells during stroke recovery [63, 76]. Increasing CBF through tPBM treatment could ensure that ischemic or damaged areas of the brain are provided with oxygen and nutrients, thus removing toxins byproducts and inflammatory mediators, reducing neuroinflammation and creating a favorable environment for neuronal repair and regeneration. Additionally, tPBM engages the neurovascular coupling mechanism, supporting long‐term synaptic plasticity and neuroplasticity.

## 4 tPBM Therapy in Human Clinical Trials

### 4.1 tPBM Treatments for Acute Ischemic Stroke: NeuroThera Effectiveness and Safety Trials (NESTs)

The first human trial of transcranial laser therapy (TLT) was the NeuroThera Effectiveness and Safety Trial‐1 (NEST‐1), a prospective, double‐blind, sham‐controlled trial that enrolled 120 stroke participants [17]. NEST‐1 delivered 808 nm wavelength light for 2 min to each of 20 predetermined locations on the scalp within 24 h of stroke symptom onset and found that the TLT group demonstrated significantly greater improvements in the primary outcome measure of complete recovery on the National Institutes of Health Stroke Scale (NIHSS) at day 90, compared with the sham‐treatment group. Importantly, the NEST‐1 also demonstrated the safety of TLT, as there were no significant between‐group differences in mortality rates or numbers of serious adverse events. The following NEST‐2 recruited 660 stroke participants with more severe strokes [71], but did not demonstrate statistically significant improvements in the primary outcome of the modified Rankin Scale (mRS) score, though post hoc analyses suggested a meaningful benefit in the subgroup with moderate to moderately severe strokes. A final pooled analysis of 778 participants revealed that the TLT group had a significantly higher success rate the on 90‐day mRS score [77]. Subgroup analyses revealed that moderate strokes and treatment 12 h after stroke onset predicted better response, suggesting delayed treatment may be more effective than early treatment. Subsequently, the large NEST‐3 was terminated early after a futility analysis of 566 participants showed no clinical difference in the primary 90‐day mRS score between the TLT group and sham‐treatment group [78]. The concurrent trial evaluating the safety of tPA plus TLT was also halted prematurely [79]. Additionally, infarct volume analyses had mixed results, with the NEST‐2 study showing no reduction in overall infarct volume or cortical infarct volume from tPBM within 24 h [80], but other research finding that tPBM within 72 h with frequent subsequent treatments reduced infarct volume and improved NIHSS scores [81], highlighting the complexity of stroke treatment.

In summary, the positive outcomes in NEST‐1 and the signal toward efficacy in NEST‐2 provided evidence of the potential benefits of tPBM treatment for acute ischemic stroke (Table 2). However, the negative results from the NEST‐3 are not entirely surprising, given the inherent challenges in acute stroke treatment. Further research and refinement of trial designs are needed to fully understand the potential benefits and limitations of tPBM for the treatment of stroke.

**TABLE 2:** Summary of t‐PBM studies on stroke in human trials.

Authors	Study design	Participants, age, number	Parameters of light	Onset of stroke	Irradiation sites	Duration time	Evaluation tools	Outcomes	SAEs
Lampl 2007 [17], NEST‐1	A prospective, intention‐to‐treat, multicenter, international, double‐blind, trial	120 ischemic stroke patients (79 TLT vs. 41 sham), age 40–85 years, NIHSS score 7–22	Laser, 808 nm, 1 J/cm^2^ of energy (NeuroThera Laser System, PhotoThera Inc.)	Within 24 h from stroke onset	20 predetermined locations, over the entire surface of the cortex regardless of stroke location	2 min at each site	NIHSS, mRS, Barthel Index, and Glasgow Outcome Scale at 90 days	NILT initiated within 24 h of ischemic stroke onset showed initial safety and effectiveness, as measured by improved NIHSS and mRS scores	Mortality rates and SAEs did not differ between groups
Zivin 2009 [71], NEST‐2	A double‐blind, randomized study	660 ischemic stroke patients (331 TLT vs. 327 sham), age 40–90 years, NIHSS score 7–22, without tPA and hemorrhagic infarct	808 nm, (NeuroThera Laser System, PhotoThera Inc.)	Within 24 h from stroke onset	20 predetermined locations	2 min at each site	mRS at 90 days	TLT within 24 h was safe but did not meet formal statistical significance for efficacy. However, predefined analyses showed a favorable trend, consistent with NEST‐1	Mortality rates and SAEs did not differ between groups
Huisa 2013 [77]	A pooled analysis	A total of 778 patients from NEST‐1 and NEST‐2	—	—	—	—	mRS 0–2 at 90 days	TLT had a significantly higher 90‐day mRS success rate compared to sham. Moderate strokes were identified as a predictor of better treatment response	—
Kasner 2013 [80]	—	640 subjects from NEST‐2, had scans on day 5 (576 CT, 64 MRI)	—	—	—	—	Infarct volumes by CT or MRI	TLT was not associated with a reduction in overall or cortical infarct volume as measured on CT in the subacute phase	—
Hacke 2014 [78], NEST‐3	A double‐blind, randomized, sham‐controlled, parallel group, multicenter trial	630 acute ischemic stroke patients (316 TLT vs. 314 sham), age 40–80 years, NIHSS score 7–17, without tPA and hemorrhagic infarct	808 nm (NeuroThera Laser System, PhotoThera Inc.)	Within 24 h from stroke onset	20 predetermined sites	2 min at each site	mRS 0–2 at 90 days	TLT showed no efficacy for the treatment of acute ischemic stroke. When combined with previous study data, there was no net benefit from TLT	SAEs did not differ between groups
Hemmen 2014 [79]	StELLAR, a small exploratory study	12 subjects (7 received tPA only, 5 tPA+ TLT), age 40–80, NIHSS score 7–17	808 nm (NeuroThera Laser System, PhotoThera Inc.)	Within 24 h from stroke onset	20 predetermined sites	2 min at each site	Rate of intracranial hemorrhages at 36 h and mRS 0–1 at 90 days	TLT was well tolerated in combination with IV tPA. Good 90‐day outcome in 3 versus 2 patients (42.9 vs. 40%, NS)	No ICH, SAEs were not different between groups
Naeser 2020 [82]	Case series report	Six people with aphasia, age 46–49 years	LED, 633 nm and 870 nm, 22.48 cm^2^, 22.2 mW/cm^2^. 500 mW (Model 1100 LED cluster head, MedX Health, Toronto)	2–18 years after single left hemisphere stroke	(1) Bilateral and midline placements including both L and R SMAs at vertex; (2) Only LH ipsilesional LED placements; (3) only LH, plus one midline cortical node of DMN (mPFC) using 13 J/cm^2^; (4) only LH, plus two midline cortical nodes of DMN (mPFC and precuneus) using 26 J/cm^2^	3 times/week, for 6 weeks	Language testing, rs‐fcMRI	NIR photons can affect surface brain cortex areas subjacent to where LEDs are applied on the scalp. Improved naming ability was present with optimal ipsilesional plus two midline DMN node placement	The pulsed rates were complete safe, even for patients with a history of seizures, and were on antiseizure medications. No seizures occurred
Estrada‐Rojas 2023 [18]	Case report	A 38‐year‐old female, with a stroke on the left side of the brain	(1) An LED cluster (630 nm, 660 nm, and 850 nm), power density of 200 mW/cm^2^, energy density of 12 J/cm^2^ per min; (2) an LED helmet (810 nm, power, 15 W; fluence, 28.8 J/cm^2^; and irradiance, 24 mW/cm^2^)	5 months poststroke	(1) Left side of the scalp, primarily along the Sylvian fissure language areas, at eight target areas; (2) an LED tPBM helmet applied to the scalp	45 min sessions, 2 times a week, for a total of 30 sessions in 5 months	Language testing	A combination of speech‐language therapy plus tPBM within a treatment session where tPBM is first applied to the left hemisphere language areas only, and then to the whole head during simultaneous speech‐language therapy was highly beneficial	—
Paolillo 2023 [83]	A randomized and placebo‐controlled study	Hemiplegia after stroke (more than 12 months from onset), age 35–75 years	Laser, 660 nm, 808 nm, 980 nm, average power of 720 mW/min, 43.2 J energy	More than 12 months from onset, 5 min after NMES	15 regions for all head	1 min per region, once a week, for 12 weeks	Grip strength, TUG, modified Ashworth Scale, VAS, MMSE, QoL	Laser and NMES treatments improved cognitive function, pain relief, manual dexterity, social–emotional health, and quality of life	No adverse effects were observed

Abbreviations: CT, computed tomography; DMN, the default mode network; LH, left hemisphere; MMSE, the mini‐mental state examination; mPFC, medial prefrontal cortex; MRI, magnetic resonance imaging; mRS, modified Rankin scale; NEST, NeuroThera effectiveness and safety trials; NIHSS, National Institutes of Health Stroke Scale; NMES, neuromuscular and muscular electrical stimulation; QoL, quality of life; rs‐fcMRI, resting‐state functional‐connectivity MRI; SAEs, serious adverse events; SMA, the supplementary motor area; TUG, timed up and go test; VAS, visual analog scale.

### 4.2 tPBM Treatment of Chronic Ischemic Stroke

A case study involving post‐stroke patients with chronic left hemisphere infarction and aphasia applied LED clusters three times per week for 6 weeks [82]. This tPBM treatment increased functional connectivity within neural networks and positively affected cortical surface areas, thereby enhancing neuromodulation. In addition, simultaneous irradiation of the ipsilateral language cortex plus two midline default mode network nodes (the medial prefrontal cortex and praecuneus) emerged as the most effective protocol for improving naming ability and enhancing impaired functional connectivity. Similarly, a combination of tPBM to left hemisphere language areas followed by whole‐head tPBM concurrent with speech‐language therapy led to significant improvements in speech rate, utterance length, and grammatical complexity compared to speech‐language therapy alone [18]. These findings suggest that a targeted tPBM approach, such as by targeting left‐hemisphere language network areas, to priming affected neural networks may enhance the benefits of tPBM combined with rehabilitation. Studies have also found that tPBM combined with other therapies can enhance stroke rehabilitation outcomes. A trial using tPBM on 15 regions across the entire head combined with neuromuscular electrical stimulation once a week for 12 weeks improved manual dexterity and motor function in chronic stroke participants [83]. Single sessions of tPBM to the left primary motor cortex increased finger tapping in healthy participants, and high‐dose tPBM was shown to instantly upregulate corticomotor excitability [72]. These findings indicate that tPBM has potential as a noninvasive neuromodulation therapy to alleviate aphasia and enhance motor recovery in chronic stroke rehabilitation.

Regarding the safety of tPBM, clinical trials have found no significant differences in adverse events like skin burns, and mortality rates, between tPBM treatment and control groups. Animal studies also show no major adverse events are associated with tPBM therapy. However, the potential cumulative thermal effects of prolonged or multiple tPBM sessions have not been well‐established due to limitations in previous trial designs.

## 5 Translational Gaps and Possible Explanations

The conclusions from the NESTs and other studies using different protocols are inconsistent, and the efficiency of tPBM to enhance the recovery of speech‐language function in chronic stroke has only been reported in a few case studies. Several reasons have been proposed for the lack of reproducible efficacy in the NESTs. First, there are challenges in translating animal models into human trials. For example, the TLT used in the NESTs was developed without optimization in multiple species or rigorous efficacy testing across translational animal models [84]. The heterogeneity of human stroke populations is also higher than that of animal models, which can contribute to variability in treatment outcomes. Additionally, for light to effectively reach brain tissue, it must penetrate at least 8 mm to account for the thickness of the human skull and dura [85]. Although studies indicate that wavelengths such as 808 nm can penetrate much deeper, reaching depths of 40–50 mm under optimal conditions [86]. Despite this capability, the complex tissue interfaces in humans also cause significant scattering, limiting photonic energy delivery to affected cortical and subcortical structures.

Second, the optimal dosimetry parameters to traverse the skull and penetrate brain tissue were not determined. For instance, the NEST‐1 specified a cortical surface fluence of 1 J/cm^2^, but the NEST‐2 and NEST‐3 did not provide details on the power density used. It is possible that the power density was insufficient to adequately penetrate the human skull and activate the biological mechanisms necessary for neuroprotection and recovery. In efforts to solve this problem, higher doses have been tested, showing that fluences of up to 120 J/cm^2^, which was 25–100 times greater than that used in the NESTs, could significantly increase cerebral metabolism and blood oxygen supply [87], and cognitive function improvements in neurological conditions [88]. NIR LEDs irradiating the scalp for 20 min in patients with TBI, delivering an incident light fluence of approximately 43 J/cm^2^, also demonstrating feasibility and safety [20, 89]. Thus, the low‐power‐density continuous‐wave (CW) mode regimen used in the NESTs may not be optimal for treating acute ischemic stroke. It has been suggested that tissue penetration can be maximized and tissue heating can be minimized by using pulsed‐wave (PW) mode rather than CW mode. PW light, with quench periods following an active pulse, allows higher power to reach deep tissues without excessive heating of surface tissues when compared with CW light at the same energy density [90, 91]. As a result, PW light is preferred for delivering high power densities to target tissues deeper than 5 cm, particularly in transcranial therapy requiring deep tissue penetration without concomitant thermal damage [92]. Researchers found PW mode increased cortical ATP concentrations by 157%–221%, versus only 41% with CW mode, showing PW is optimal for mitochondrial stimulation and potentially preserving tissue in ischemic penumbrae [50].

**FIGURE 2:**
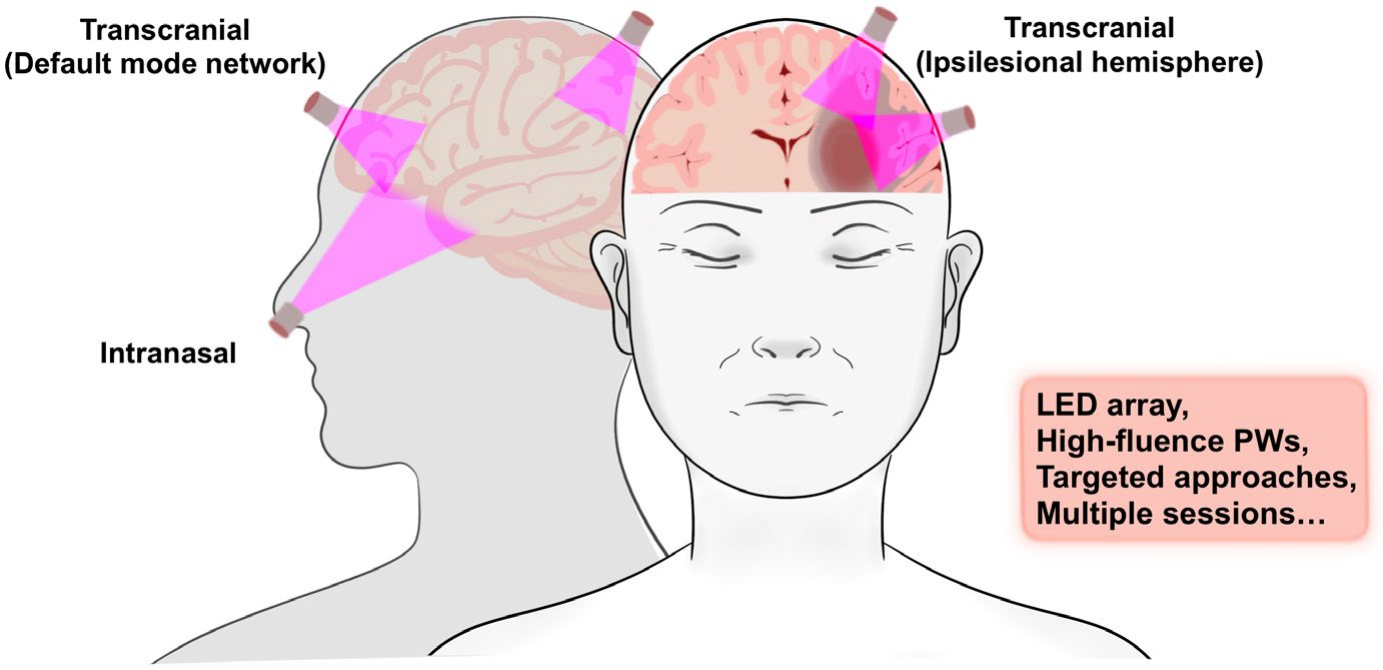
Several potential tPBM delivery approaches for future stroke research. This could involve using safer LED array devices instead of lasers, as well as applying high‐fluence PW light rather than CW. Combining targeted application to specific brain regions, such as the ipsilateral side and default mode network, with multiple treatment sessions may enhance tPBM's neuroprotective and restorative effects. Additionally, intranasal tPBM using light‐emitting nasal devices could provide efficient near‐infrared light delivery to deep brain structures.

Third, the protocol used for stroke treatment is an important consideration. Animal study showed that a single tPBM treatment can promote behavioral recovery for 21 days [46], and the NESTs demonstrated the effectiveness of a single dose by measuring clinical efficacy 90 days later [17]. However, a single treatment may be insufficient for some brain areas, thereby necessitating repeated treatments. Studies have shown that delivering large total doses in a single session may yield less favorable outcomes than the same doses over multiple sessions [93]. Furthermore, a focused tPBM approach may be more effective than the broad application used in the NESTs, where only a few of the 20 skull sites were likely adjacent to penumbral tissue, and a 2‐min application might have been insufficient. Findings suggest that a more targeted tPBM approach to only the ipsilateral side and default mode network nodes with multiple tPBM sessions [18, 82] could be more effective than the NESTs approach (Figure 2).

## 6 Conclusion and Future Perspective

Currently, research on tPBM therapy remains in its early stages, but the solid mechanistic theory and its potential to be a non‐invasive, cost‐effective, and safe long‐term treatment option for neurological and psychological conditions, make it highly appealing. There is emerging enthusiasm for tPBM treatments to positively influence neuronal activity and achieve favorable outcomes in patients with neurological conditions like stroke, TBI, Parkinson's, Alzheimer's, and depression. Proof‐of‐concept studies have shown tPBM can improve motor and non‐motor symptoms, such as enhancing cognitive scores, verbal memory, and sleep quality, and alleviating post‐traumatic stress in TBI patients [42]. Preliminary research also indicates tPBM may improve sleep and reduce anxiety in patients with generalized anxiety disorders [94]. However, these results should be interpreted cautiously due to small sample sizes and lack of control groups. More rigorous double‐blind studies are needed to confirm the effectiveness of tPBM for neurological diseases.

The continual development of tPBM equipment is crucial. Initially, tPBM treatment involved irradiating the forehead with laser light due to its good penetration abilities [83, 95]. More recently, the introduction of inexpensive LED arrays has facilitated the creation of light‐emitting helmets [96]. Additionally, a NIR LED noseclip has also been developed to deliver photons directly to the olfactory bulbs and enable therapeutic doses of light to penetrate the brain [97, 98, 99]. It is suggested that the intranasal approach seems to be more effective in transmitting light to deep brain structures, such as the limbic system and prefrontal lobe, and claimed to improve the symptoms of AD, PD, and depression [100, 101]. Another expected advancement is the use of organic LEDs, which emit light when exposed to an electric current [25, 102], further expanding the potential applications by targeting specific brain structures and enabling various stimulation modes.

Recent research has demonstrated that tPBM with NIR‐II (1000–1700 nm) laser light at a wavelength of 1064 nm significantly enhances CBF in mouse models of ischemic stroke, leading to reduced infarct volumes and enhanced neurological function [70]. Additionally, NIR‐II laser light at a wavelength of 1267 nm has also been shown to facilitate rapid recovery in newborn rats following intraventricular hemorrhage due to improvements in lymphatic drainage and clearing functions [103]. These findings exemplify the potential of tPBM in ameliorating stroke injury and indicate that there remains considerable opportunity for further research to optimize treatment protocols and explore its applications across various neurological conditions.

In conclusion, tPBM holds great promise as a therapeutic tool for stroke and other neurological conditions. However, a rigorous re‐evaluation of tPBM is required, with an emphasis on enhancing device technology, optimizing treatment parameters and protocols, and conducting thorough testing in both animal models and human clinical studies.

## Author Contributions

S.L. and S.S.M.N. designed and wrote the manuscript. T.W.L.W. and S.S.M.N. revised the manuscript. S.S.M.N. gave constructive advice and participated in proofreading this paper. All authors contributed to the article and approved the submitted version.

## Conflicts of Interest

The authors declare no conflicts of interest.

## Data Availability

Data sharing is not applicable to this article as no new data were created or analyzed in this study.

## References

[1] A. Abijo , C. Y. Lee , C. Y. Huang , P. C. Ho , and K. J. Tsai , “The Beneficial Role of Photobiomodulation in Neurodegenerative Diseases,” Biomedicine 11, no. 7 (2023): 1828. 10.3390/biomedicines11071828 PMC10377111 37509468

[2] K. Montazeri , M. Farhadi , R. Fekrazad , Z. Akbarnejad , S. Chaibakhsh , and S. Mahmoudian , “Transcranial Photobiomodulation in the Management of Brain Disorders,” Journal of Photochemistry and Photobiology. B 221 (2021): 112207. 10.1016/j.jphotobiol.2021.112207 34119804

[3] C. Thunshelle and M. R. Hamblin , “Transcranial Low‐Level Laser (Light) Therapy for Brain Injury,” Photomedicine and Laser Surgery 34, no. 12 (2016): 587–598. 28001759 10.1089/pho.2015.4051 PMC5180077

[4] W. F. Vieira , D. V. Iosifescu , K. M. McEachern , M. Gersten , and P. Cassano , “Photobiomodulation: An Emerging Treatment Modality for Depression,” Psychiatric Clinics of North America 46, no. 2 (2023): 331–348. 37149348 10.1016/j.psc.2023.02.013

[5] J. T. Hashmi , Y. Y. Huang , B. Z. Osmani , S. K. Sharma , M. A. Naeser , and M. R. Hamblin , “Role of Low‐Level Laser Therapy in Neurorehabilitation,” PM & R: Journal of Injury, Function, and Rehabilitation 2, no. 12 Suppl 2 (2010): S292–S305. 10.1016/j.pmrj.2010.10.013 PMC3065857 21172691

[6] M. R. Hamblin , “Shining Light on the Head: Photobiomodulation for Brain Disorders,” BBA Clinical 6 (2016): 113–124. 27752476 10.1016/j.bbacli.2016.09.002 PMC5066074

[7] H. Serrage , V. Heiskanen , W. M. Palin , et al., “Under the Spotlight: Mechanisms of Photobiomodulation Concentrating on Blue and Green Light,” Photochemical & Photobiological Sciences 18, no. 8 (2019): 1877–1909. 31183484 10.1039/c9pp00089e PMC6685747

[8] C. Ash , M. Dubec , K. Donne , and T. Bashford , “Effect of Wavelength and Beam Width on Penetration in Light‐Tissue Interaction Using Computational Methods,” Lasers in Medical Science 32, no. 8 (2017): 1909–1918. 28900751 10.1007/s10103-017-2317-4 PMC5653719

[9] N. Tripodi , F. Sidiroglou , S. Fraser , et al., “The Effects of Polarized Photobiomodulation on Cellular Viability, Proliferation, Mitochondrial Membrane Potential and Apoptosis in Human Fibroblasts: Potential Applications to Wound Healing,” Journal of Photochemistry and Photobiology. B Biology 236 (2022): 112574. 36179581 10.1016/j.jphotobiol.2022.112574

[10] S. Navarro‐Ledesma , J. Carroll , P. Burton , and G. M. Ana , “Short‐Term Effects of Whole‐Body Photobiomodulation on Pain, Quality of Life and Psychological Factors in a Population Suffering From Fibromyalgia: A Triple‐Blinded Randomised Clinical Trial,” Pain and Therapy 12, no. 1 (2023): 225–239. 36369323 10.1007/s40122-022-00450-5 PMC9845459

[11] N. Tripodi , J. Feehan , M. Husaric , F. Sidiroglou , and V. Apostolopoulos , “The Effect of Low‐Level Red and Near‐Infrared Photobiomodulation on Pain and Function in Tendinopathy: A Systematic Review and Meta‐Analysis of Randomized Control Trials,” BMC Sports Science, Medicine and Rehabilitation 13, no. 1 (2021): 91. 10.1186/s13102-021-00306-z PMC8364035 34391447

[12] W. T. Luo , C. J. Lee , K. W. Tam , and T. W. Huang , “Effects of Low‐Level Laser Therapy on Muscular Performance and Soreness Recovery in Athletes: A Meta‐Analysis of Randomized Controlled Trials,” Sports Health 14, no. 5 (2022): 687–693. 34428975 10.1177/19417381211039766 PMC9460079

[13] S. Oliveira , R. Andrade , C. Valente , et al., “Effectiveness of Photobiomodulation in Reducing Pain and Disability in Patients With Knee Osteoarthritis: A Systematic Review With Meta‐Analysis,” Physical Therapy 104 (2024): pzae073. 38775202 10.1093/ptj/pzae073

[14] B. Shen , Y. Zhou , D. Wu , and J. Liu , “Efficacy of Photobiomodulation Therapy in the Management of Oral Mucositis in Patients With Head and Neck Cancer: A Systematic Review and Meta‐Analysis of Randomized Controlled Trials,” Head and Neck 46, no. 4 (2024): 936–950. 38265122 10.1002/hed.27655

[15] M. C. Petrellis , G. da Fonseca , P. A. de Barros , et al., “Laser‐Photobiomodulation on Experimental Cancer Pain Model in Walker Tumor‐256,” Journal of Photochemistry and Photobiology. B Biology 210 (2020): 111979. 32738748 10.1016/j.jphotobiol.2020.111979

[16] G. Onose , A. Anghelescu , D. Blendea , et al., “Cellular and Molecular Targets for Non‐Invasive, Non‐Pharmacological Therapeutic/Rehabilitative Interventions in Acute Ischemic Stroke,” International Journal of Molecular Sciences 23, no. 2 (2022): 907. 35055089 10.3390/ijms23020907 PMC8846361

[17] Y. Lampl , J. A. Zivin , M. Fisher , et al., “Infrared Laser Therapy for Ischemic Stroke: A New Treatment Strategy: Results of the NeuroThera Effectiveness and Safety Trial‐1 (NEST‐1),” Stroke 38, no. 6 (2007): 1843–1849. 17463313 10.1161/STROKEAHA.106.478230

[18] K. Estrada‐Rojas and N. P. C. Ortiz , “Increased Improvement in Speech‐Language Skills After Transcranial Photobiomodulation Plus Speech‐Language Therapy, Compared to Speech‐Language Therapy Alone: Case Report With Aphasia,” Photobiomodulation, Photomedicine, and Laser Surgery 41, no. 5 (2023): 234–240. 36999917 10.1089/photob.2022.0024 PMC10171962

[19] L. Lim , “Traumatic Brain Injury Recovery With Photobiomodulation: Cellular Mechanisms, Clinical Evidence, and Future Potential,” Cells 13, no. 5 (2024): 385. 38474349 10.3390/cells13050385 PMC10931349

[20] A. R. Stevens , M. Hadis , M. Milward , et al., “Photobiomodulation in Acute Traumatic Brain Injury: A Systematic Review and Meta‐Analysis,” Journal of Neurotrauma 40, no. 3–4 (2023): 210–227. 35698294 10.1089/neu.2022.0140

[21] Z. Huang , M. R. Hamblin , and Q. Zhang , “Photobiomodulation in Experimental Models of Alzheimer's Disease: State‐of‐The‐Art and Translational Perspectives,” Alzheimer's Research and Therapy 16, no. 1 (2024): 114. 10.1186/s13195-024-01484-x PMC11106984 38773642

[22] H. Xu , Z. Luo , R. Zhang , et al., “Exploring the Effect of Photobiomodulation and Gamma Visual Stimulation Induced by 808 nm and Visible LED in Alzheimer's Disease Mouse Model,” Journal of Photochemistry and Photobiology. B Biology 250 (2024): 112816. 38029664 10.1016/j.jphotobiol.2023.112816

[23] C. McGee , A. Liebert , B. Bicknell , et al., “A Randomized Placebo‐Controlled Study of a Transcranial Photobiomodulation Helmet in Parkinson's Disease: Post‐Hoc Analysis of Motor Outcomes,” Journal of Clinical Medicine 12, no. 8 (2023): 2846. 37109183 10.3390/jcm12082846 PMC10146323

[24] M. Yang , Z. Yang , P. Wang , and Z. Sun , “Current Application and Future Directions of Photobiomodulation in Central Nervous Diseases,” Neural Regeneration Research 16, no. 6 (2021): 1177–1185. 33269767 10.4103/1673-5374.300486 PMC8224127

[25] H. Ma , Y. Du , D. Xie , Z. Z. Wei , Y. Pan , and Y. Zhang , “Recent Advances in Light Energy Biotherapeutic Strategies With Photobiomodulation on Central Nervous System Disorders,” Brain Research 1822 (2024): 148615. 37783261 10.1016/j.brainres.2023.148615

[26] C. D. Anderson , A. Biffi , M. A. Nalls , et al., “Common Variants Within Oxidative Phosphorylation Genes Influence Risk of Ischemic Stroke and Intracerebral Hemorrhage,” Stroke 44, no. 3 (2013): 612–619. 23362085 10.1161/STROKEAHA.112.672089 PMC3582722

[27] T. H. Sanderson , C. A. Reynolds , R. Kumar , K. Przyklenk , and M. Huttemann , “Molecular Mechanisms of Ischemia‐Reperfusion Injury in Brain: Pivotal Role of the Mitochondrial Membrane Potential in Reactive Oxygen Species Generation,” Molecular Neurobiology 47, no. 1 (2013): 9–23. 23011809 10.1007/s12035-012-8344-z PMC3725766

[28] A. Milanlioglu , M. Aslan , H. Ozkol , V. Cilingir , M. Nuri Aydin , and S. Karadas , “Serum Antioxidant Enzymes Activities and Oxidative Stress Levels in Patients With Acute Ischemic Stroke: Influence on Neurological Status and Outcome,” Wiener Klinische Wochenschrift 128, no. 5–6 (2016): 169–174. 25854910 10.1007/s00508-015-0742-6

[29] A. Valverde , C. Hamilton , C. Moro , M. Billeres , P. Magistretti , and J. Mitrofanis , “Lights at Night: Does Photobiomodulation Improve Sleep?,” Neural Regeneration Research 18, no. 3 (2023): 474–477. 36018149 10.4103/1673-5374.350191 PMC9727457

[30] H. Joshi , P. Sinha , D. Bowers , and J. P. John , “Dose Response of Transcranial Near Infrared Light Stimulation on Brain Functional Connectivity and Cognition in Older Adults—A Randomized Comparison,” Journal of Biophotonics 17, no. 2 (2024): e202300215. 37776079 10.1002/jbio.202300215

[31] B. Bicknell , A. Liebert , and G. Herkes , “Parkinson's Disease and Photobiomodulation: Potential for Treatment,” Journal of Personalized Medicine 14, no. 1 (2024): 112. 38276234 10.3390/jpm14010112 PMC10819946

[32] G. M. Dmochowski , A. D. Shereen , D. Berisha , and J. P. Dmochowski , “Near‐Infrared Light Increases Functional Connectivity With a Non‐Thermal Mechanism,” Cerebral Cortex Communications 1, no. 1 (2020): tgaa004. 34296085 10.1093/texcom/tgaa004 PMC8152883

[33] A. H. Ghaderi , A. Jahan , F. Akrami , and S. M. Moghadam , “Transcranial Photobiomodulation Changes Topology, Synchronizability, and Complexity of Resting State Brain Networks,” Journal of Neural Engineering 18, no. 4 (2021): 46048. 10.1088/1741-2552/abf97c 33873167

[34] A. Liebert , B. Bicknell , E. L. Laakso , et al., “Improvements in Clinical Signs of Parkinson's Disease Using Photobiomodulation: A Prospective Proof‐of‐Concept Study,” BMC Neurology 21, no. 1 (2021): 256. 34215216 10.1186/s12883-021-02248-y PMC8249215

[35] S. Purushothuman , C. Nandasena , D. M. Johnstone , J. Stone , and J. Mitrofanis , “The Impact of Near‐Infrared Light on Dopaminergic Cell Survival in a Transgenic Mouse Model of Parkinsonism,” Brain Research 1535 (2013): 61–70. 23998985 10.1016/j.brainres.2013.08.047

[36] N. A. Zhevago and K. A. Samoilova , “Pro‐ and Anti‐Inflammatory Cytokine Content in Human Peripheral Blood After Its Transcutaneous (In Vivo) and Direct (In Vitro) Irradiation With Polychromatic Visible and Infrared Light,” Photomedicine and Laser Surgery 24, no. 2 (2006): 129–139. 16706691 10.1089/pho.2006.24.129

[37] Z. H. Zhang , T. Y. Wu , C. Ju , et al., “Photobiomodulation Increases M2‐Type Polarization of Macrophages by Inhibiting Versican Production After Spinal Cord Injury,” Molecular Neurobiology 61 (2024): 6950–6967. 38363534 10.1007/s12035-024-03980-5

[38] H. I. Lee , S. W. Lee , N. G. Kim , et al., “Low‐Level Light Emitting Diode Therapy Promotes Long‐Term Functional Recovery After Experimental Stroke in Mice,” Journal of Biophotonics 10, no. 12 (2017): 1761–1771. 28464523 10.1002/jbio.201700038

[39] H. I. Lee , S. W. Lee , N. G. Kim , et al., “Low‐Level Light Emitting Diode (LED) Therapy Suppresses Inflammasome‐Mediated Brain Damage in Experimental Ischemic Stroke,” Journal of Biophotonics 10, no. 11 (2017): 1502–1513. 28164443 10.1002/jbio.201600244

[40] R. Wang , Y. Dong , Y. Lu , W. Zhang , D. W. Brann , and Q. Zhang , “Photobiomodulation for Global Cerebral Ischemia: Targeting Mitochondrial Dynamics and Functions,” Molecular Neurobiology 56, no. 3 (2019): 1852–1869. 29951942 10.1007/s12035-018-1191-9 PMC6310117

[41] D. D. S. Vogel , N. N. Ortiz‐Villatoro , N. S. Araújo , et al., “Transcranial Low‐Level Laser Therapy in an In Vivo Model of Stroke: Relevance to the Brain Infarct, Microglia Activation and Neuroinflammation,” Journal of Biophotonics 14, no. 6 (2021): e202000500. 33580734 10.1002/jbio.202000500

[42] T. L. Lee , Z. Ding , and A. S. Chan , “Can Transcranial Photobiomodulation Improve Cognitive Function? A Systematic Review of Human Studies,” Ageing Research Reviews 83 (2023): 101786. 36371017 10.1016/j.arr.2022.101786

[43] D. D. S. Vogel , N. N. Ortiz‐Villatoro , L. de Freitas , et al., “Repetitive Transcranial Photobiomodulation but Not Long‐Term Omega‐3 Intake Reduces Epileptiform Discharges in Rats With Stroke‐Induced Epilepsy,” Journal of Biophotonics 14, no. 1 (2021): e202000287. 32888387 10.1002/jbio.202000287

[44] M. C. Leung , S. C. Lo , F. K. Siu , and K. F. So , “Treatment of Experimentally Induced Transient Cerebral Ischemia With Low Energy Laser Inhibits Nitric Oxide Synthase Activity and Up‐Regulates the Expression of Transforming Growth Factor‐Beta 1,” Lasers in Surgery and Medicine 31, no. 4 (2002): 283–288. 12355575 10.1002/lsm.10096

[45] P. A. Lapchak , J. Wei , and J. A. Zivin , “Transcranial Infrared Laser Therapy Improves Clinical Rating Scores After Embolic Strokes in Rabbits,” Stroke 35, no. 8 (2004): 1985–1988. 15155955 10.1161/01.STR.0000131808.69640.b7

[46] L. DeTaboada , S. Ilic , S. Leichliter‐Martha , U. Oron , A. Oron , and J. Streeter , “Transcranial Application of Low‐Energy Laser Irradiation Improves Neurological Deficits in Rats Following Acute Stroke,” Lasers in Surgery and Medicine 38, no. 1 (2006): 70–73. 16444697 10.1002/lsm.20256

[47] A. Oron , U. Oron , J. Chen , et al., “Low‐Level Laser Therapy Applied Transcranially to Rats After Induction of Stroke Significantly Reduces Long‐Term Neurological Deficits,” Stroke 37, no. 10 (2006): 2620–2624. 16946145 10.1161/01.STR.0000242775.14642.b8

[48] P. A. Lapchak , K. F. Salgado , C. H. Chao , and J. A. Zivin , “Transcranial Near‐Infrared Light Therapy Improves Motor Function Following Embolic Strokes in Rabbits: An Extended Therapeutic Window Study Using Continuous and Pulse Frequency Delivery Modes,” Neuroscience 148, no. 4 (2007): 907–914. 17693028 10.1016/j.neuroscience.2007.07.002

[49] P. A. Lapchak , M. K. Han , K. F. Salgado , J. Streeter , and J. A. Zivin , “Safety Profile of Transcranial Near‐Infrared Laser Therapy Administered in Combination With Thrombolytic Therapy to Embolized Rabbits,” Stroke 39, no. 11 (2008): 3073–3078. 18687999 10.1161/STROKEAHA.108.516393

[50] P. A. Lapchak and L. De Taboada , “Transcranial Near Infrared Laser Treatment (NILT) Increases Cortical Adenosine‐5′‐Triphosphate (ATP) Content Following Embolic Strokes in Rabbits,” Brain Research 1306 (2010): 100–105. 19837048 10.1016/j.brainres.2009.10.022

[51] Y. Uozumi , H. Nawashiro , S. Sato , S. Kawauchi , K. Shima , and M. Kikuchi , “Targeted Increase in Cerebral Blood Flow by Transcranial Near‐Infrared Laser Irradiation,” Lasers in Surgery and Medicine 42, no. 6 (2010): 566–576. 20662034 10.1002/lsm.20938

[52] K. K. Yip , S. C. Lo , M. C. Leung , K. F. So , C. Y. Tang , and D. M. Poon , “The Effect of Low‐Energy Laser Irradiation on Apoptotic Factors Following Experimentally Induced Transient Cerebral Ischemia,” Neuroscience 190 (2011): 301–306. 21712070 10.1016/j.neuroscience.2011.06.022

[53] B. N. Huisa , Y. M. Chen , B. C. Meyer , G. M. Tafreshi , and J. A. Zivin , “Incremental Treatments With Laser Therapy Augments Good Behavioral Outcome in the Rabbit Small Clot Embolic Stroke Model,” Lasers in Medical Science 28, no. 4 (2013): 1085–1089. 22945539 10.1007/s10103-012-1193-1 PMC3538923

[54] P. A. Lapchak and P. D. Boitano , “A Novel Method to Promote Behavioral Improvement and Enhance Mitochondrial Function Following an Embolic Stroke,” Brain Research 1646 (2016): 125–131. 27180104 10.1016/j.brainres.2016.04.039

[55] H. I. Lee , J. H. Park , M. Y. Park , et al., “Pre‐Conditioning With Transcranial Low‐Level Light Therapy Reduces Neuroinflammation and Protects Blood‐Brain Barrier After Focal Cerebral Ischemia in Mice,” Restorative Neurology and Neuroscience 34, no. 2 (2016): 201–214. 26889965 10.3233/RNN-150559

[56] D. M. Meyer , Y. M. Chen , and J. A. Zivin , “Dose‐Finding Study of Phototherapy on Stroke Outcome in a Rabbit Model of Ischemic Stroke,” Neuroscience Letters 630 (2016): 254–258. 27345389 10.1016/j.neulet.2016.06.038

[57] H. I. Lee , S. W. Lee , S. Y. Kim , et al., “Pretreatment With Light‐Emitting Diode Therapy Reduces Ischemic Brain Injury in Mice Through Endothelial Nitric Oxide Synthase‐Dependent Mechanisms,” Biochemical and Biophysical Research Communications 486, no. 4 (2017): 945–950. 28347821 10.1016/j.bbrc.2017.03.131

[58] T. H. Sanderson , J. M. Wider , I. Lee , et al., “Inhibitory Modulation of Cytochrome c Oxidase Activity With Specific Near‐Infrared Light Wavelengths Attenuates Brain Ischemia/Reperfusion Injury,” Scientific Reports 8 (2018): 3481. 29472564 10.1038/s41598-018-21869-x PMC5823933

[59] L. D. Yang , D. Tucker , Y. Dong , et al., “Photobiomodulation Therapy Promotes Neurogenesis by Improving Post‐Stroke Local Microenvironment and Stimulating Neuroprogenitor Cells,” Experimental Neurology 299 (2018): 86–96. 29056360 10.1016/j.expneurol.2017.10.013 PMC5723531

[60] B. Argibay , F. Campos , M. Perez‐Mato , et al., “Light‐Emitting Diode Photobiomodulation After Cerebra Ischemia,” Frontiers in Neurology 10 (2019): 911. 31507516 10.3389/fneur.2019.00911 PMC6713875

[61] E. G. D. Fonseca , A. Pedroso , D. Neuls , et al., “Study of Transcranial Therapy 904 nm in Experimental Model of Stroke,” Lasers in Medical Science 34, no. 8 (2019): 1619–1625. 30826952 10.1007/s10103-019-02758-9

[62] F. Salehpour , F. Farajdokht , J. Mahmoudi , et al., “Photobiomodulation and Coenzyme Q10 Treatments Attenuate Cognitive Impairment Associated With Model of Transient Global Brain Ischemia in Artificially Aged Mice,” Frontiers in Cellular Neuroscience 13 (2019): 1–17. 30983970 10.3389/fncel.2019.00074 PMC6434313

[63] A. Vahabzadeh‐Hagh , T. J. McCarthy , L. De Taboada , et al., “Near Infrared Light Amplifies Endothelial Progenitor Cell Accumulation After Stroke,” Conditioning Medicine 2, no. 4 (2019): 170–177. 34291201 PMC8291201

[64] C. D. Strubakos , M. Malik , J. M. Wider , et al., “Non‐invasive Treatment With Near‐Infrared Light: A Novel Mechanisms‐Based Strategy That Evokes Sustained Reduction in Brain Injury After Stroke,” Journal of Cerebral Blood Flow and Metabolism 40, no. 4 (2020): 833–844. 31112450 10.1177/0271678X19845149 PMC7168789

[65] S. Guo , R. Wang , J. Hu , et al., “Photobiomodulation Promotes Hippocampal CA1 NSC Differentiation Toward Neurons and Facilitates Cognitive Function Recovery Involving NLRP3 Inflammasome Mitigation Following Global Cerebral Ischemia,” Frontiers in Cellular Neuroscience 15 (2021): 731855. 34489645 10.3389/fncel.2021.731855 PMC8417562

[66] H. Kim , M. J. Kim , Y. W. Kwon , et al., “Benefits of a Skull‐Interfaced Flexible and Implantable Multilight Emitting Diode Array for Photobiomodulation in Ischemic Stroke,” Advanced Science 9, no. 11 (2022): e2104629. 35076161 10.1002/advs.202104629 PMC9008794

[67] Y. Feng , L. Yang , X. Ma , et al., “Photobiomodulation Treatment Inhibits Neurotoxic Astrocytic Polarization and Protects Neurons in In Vitro and In Vivo Stroke Models,” Neurochemistry International 162 (2023): 105464. 36539162 10.1016/j.neuint.2022.105464 PMC12798860

[68] R. A. Shalaby , M. M. Qureshi , M. A. Khan , et al., “Photobiomodulation Therapy Restores Olfactory Function Impaired by Photothrombosis in Mouse Olfactory Bulb,” Experimental Neurology 367 (2023): 114462. 37295546 10.1016/j.expneurol.2023.114462

[69] Y. Feng , Z. Huang , X. Ma , et al., “Activation of Testosterone‐Androgen Receptor Mediates Cerebrovascular Protection by Photobiomodulation Treatment in Photothrombosis‐Induced Stroke Rats,” CNS Neuroscience and Therapeutics 30, no. 2 (2024): e14574. 38421088 10.1111/cns.14574 PMC10851319

[70] S. Yokomizo , T. Kopp , M. Roessing , et al., “Near‐Infrared II Photobiomodulation Preconditioning Ameliorates Stroke Injury via Phosphorylation of eNOS,” Stroke 55 (2024): 1641–1649. 38572660 10.1161/STROKEAHA.123.045358 PMC11126363

[71] J. A. Zivin , G. W. Albers , N. Bornstein , et al., “Effectiveness and Safety of Transcranial Laser Therapy for Acute Ischemic Stroke,” Stroke 40, no. 4 (2009): 1359–1364. 19233936 10.1161/STROKEAHA.109.547547

[72] Y. L. Kuo and B. Kim , “High Dosage Transcranial Photobiomodulation Increases Corticomotor Excitability in an Individual With Chronic Stroke: A Case Report,” Brain Stimulation 16, no. 1 (2023): 354.

[73] B. L. Marques , O. C. Oliveira‐Lima , G. A. Carvalho , et al., “Neurobiology of Glycine Transporters: From Molecules to Behavior,” Neuroscience and Biobehavioral Reviews 118 (2020): 97–110. 32712279 10.1016/j.neubiorev.2020.07.025

[74] D. H. Choi , J. H. Lim , K. H. Lee , et al., “Effect of 710‐nm Visible Light Irradiation on Neuroprotection and Immune Function After Stroke,” Neuroimmunomodulation 19, no. 5 (2012): 267–276. 22472725 10.1159/000335547

[75] K. Panbhare , R. Pandey , C. Chauhan , A. Sinha , R. Shukla , and R. K. Kaundal , “Role of NLRP3 Inflammasome in Stroke Pathobiology: Current Therapeutic Avenues and Future Perspective,” ACS Chemical Neuroscience 15, no. 1 (2024): 31–55. 38118278 10.1021/acschemneuro.3c00536

[76] J. Li , Z. Wang , J. Li , H. Zhao , and Q. Ma , “HMGB1: A New Target for Ischemic Stroke and Hemorrhagic Transformation,” Translational Stroke Research (2024). 10.1007/s12975-024-01258-5 PMC12045843 38740617

[77] B. N. Huisa , A. B. Stemer , M. G. Walker , et al., “Transcranial Laser Therapy for Acute Ischemic Stroke: A Pooled Analysis of NEST‐1 and NEST‐2,” International Journal of Stroke 8, no. 5 (2013): 315–320. 22299818 10.1111/j.1747-4949.2011.00754.x PMC3345315

[78] W. Hacke , “Transcranial Laser Therapy in Acute Stroke Treatment: Results of Neurothera Effectiveness and Safety Trial 3, a Phase III Clinical End Point Device Trial,” Stroke 45, no. 12 (2014): 3187–3193. 25293665 10.1161/STROKEAHA.114.005795

[79] T. Hemmen , R. Raman , S. Sen , et al., “Safety of TPA + Transcranial Emission of Low‐Energy Lasers for Acute Stroke Recovery‐Results,” Neurology 82, no. 10 (2014): 4.

[80] S. E. Kasner , D. Z. Rose , A. Skokan , et al., “Transcranial Laser Therapy and Infarct Volume,” Stroke 44, no. 7 (2013): 2025–2027. 23660846 10.1161/STROKEAHA.113.000870

[81] J. Moreira , “Laser Photobiomodulation: A Novel Treatment for Acute Stroke?,” Journal of Neuroimaging 30, no. 3 (2020): 381.

[82] M. A. Naeser , M. D. Ho , P. I. Martin , M. R. Hamblin , and B. B. Koo , “Increased Functional Connectivity Within Intrinsic Neural Networks in Chronic Stroke Following Treatment With Red/Near‐Infrared Transcranial Photobiomodulation: Case Series With Improved Naming in Aphasia,” Photobiomodulation, Photomedicine, and Laser Surgery 38, no. 2 (2020): 115–131. 31621498 10.1089/photob.2019.4630

[83] F. R. Paolillo , G. A. A. Luccas , N. A. Parizotto , A. R. Paolillo , J. C. D. Neto , and V. S. Bagnato , “The Effects of Transcranial Laser Photobiomodulation and Neuromuscular Electrical Stimulation in the Treatment of Post‐Stroke Dysfunctions,” Journal of Biophotonics 16, no. 4 (2023): e202200260. 36520347 10.1002/jbio.202200260

[84] P. A. Lapchak and P. D. Boitano , “Transcranial Near‐Infrared Laser Therapy for Stroke: How to Recover From Futility in the NEST‐3 Clinical Trial,” Acta Neurochirurgica. Supplement 121 (2016): 7–12. 26463915 10.1007/978-3-319-18497-5_2

[85] P. A. Lapchak , “Transcranial Near‐Infrared Laser Therapy Applied to Promote Clinical Recovery in Acute and Chronic Neurodegenerative Diseases,” Expert Review of Medical Devices 9, no. 1 (2012): 71–83. 22145842 10.1586/erd.11.64 PMC3270070

[86] C. E. Tedford , S. DeLapp , S. Jacques , and J. Anders , “Quantitative Analysis of Transcranial and Intraparenchymal Light Penetration in Human Cadaver Brain Tissue,” Lasers in Surgery and Medicine 47, no. 4 (2015): 312–322. 25772014 10.1002/lsm.22343

[87] F. Gonzalez‐Lima and A. Auchter , “Protection Against Neurodegeneration With Low‐Dose Methylene Blue and Near‐Infrared Light,” Frontiers in Cellular Neuroscience 9 (2015): 179. 26029050 10.3389/fncel.2015.00179 PMC4428125

[88] F. D. S. Cardoso , F. Gonzalez‐Lima , and S. Gomes da Silva , “Photobiomodulation for the Aging Brain,” Ageing Research Reviews 70 (2021): 101415. 34325071 10.1016/j.arr.2021.101415

[89] M. G. Figueiro Longo , C. O. Tan , S. T. Chan , et al., “Effect of Transcranial Low‐Level Light Therapy vs Sham Therapy Among Patients With Moderate Traumatic Brain Injury: A Randomized Clinical Trial,” JAMA Network Open 3, no. 9 (2020): e2017337. 32926117 10.1001/jamanetworkopen.2020.17337 PMC7490644

[90] C. M. Krause , M. Pesonen , C. Haarala Bjornberg , and H. Hamalainen , “Effects of Pulsed and Continuous Wave 902 MHz Mobile Phone Exposure on Brain Oscillatory Activity During Cognitive Processing,” Bioelectromagnetics 28, no. 4 (2007): 296–308. 17203478 10.1002/bem.20300

[91] T. Ando , W. Xuan , T. Xu , et al., “Comparison of Therapeutic Effects Between Pulsed and Continuous Wave 810‐nm Wavelength Laser Irradiation for Traumatic Brain Injury in Mice,” PLoS One 6, no. 10 (2011): e26212. 22028832 10.1371/journal.pone.0026212 PMC3196530

[92] Y. Chen , L. De Taboada , M. O'Connor , S. Delapp , and J. A. Zivin , “Thermal Effects of Transcranial Near‐Infrared Laser Irradiation on Rabbit Cortex,” Neuroscience Letters 553 (2013): 99–103. 23933199 10.1016/j.neulet.2013.07.049

[93] R. Zein , W. Selting , and M. R. Hamblin , “Review of Light Parameters and Photobiomodulation Efficacy: Dive Into Complexity,” Journal of Biomedical Optics 23, no. 12 (2018): 1–17. 10.1117/1.JBO.23.12.120901 PMC8355782 30550048

[94] B. Guo , M. Zhang , W. Hao , Y. Wang , T. Zhang , and C. Liu , “Neuroinflammation Mechanisms of Neuromodulation Therapies for Anxiety and Depression,” Translational Psychiatry 13, no. 1 (2023): 5. 36624089 10.1038/s41398-022-02297-y PMC9829236

[95] M. A. Naeser , “Photobiomodulation of Pain in Carpal Tunnel Syndrome: Review of Seven Laser Therapy Studies,” Photomedicine and Laser Surgery 24, no. 2 (2006): 101–110. 16706688 10.1089/pho.2006.24.101

[96] W. W. Zhang , X. Y. Wang , Y. X. Chu , and Y. Q. Wang , “Light‐Emitting Diode Phototherapy: Pain Relief and Underlying Mechanisms,” Lasers in Medical Science 37, no. 5 (2022): 2343–2352. 35404002 10.1007/s10103-022-03540-0

[97] M. A. Naeser , P. I. Martin , M. D. Ho , et al., “Transcranial, Red/Near‐Infrared Light‐Emitting Diode Therapy to Improve Cognition in Chronic Traumatic Brain Injury,” Photomedicine and Laser Surgery 34, no. 12 (2016): 610–626. 28001756 10.1089/pho.2015.4037

[98] S. Won , J. An , H. Song , et al., “Transnasal Targeted Delivery of Therapeutics in Central Nervous System Diseases: A Narrative Review,” Frontiers in Neuroscience 17 (2023): 1137096. 37292158 10.3389/fnins.2023.1137096 PMC10246499

[99] K. C. Kosel , G. W. Van Hoesen , and J. R. West , “Olfactory Bulb Projections to the Parahippocampal Area of the Rat,” Journal of Comparative Neurology 198, no. 3 (1981): 467–482. 7240454 10.1002/cne.901980307

[100] A. E. Saltmarche , M. A. Naeser , K. F. Ho , M. R. Hamblin , and L. Lim , “Significant Improvement in Cognition in Mild to Moderately Severe Dementia Cases Treated With Transcranial Plus Intranasal Photobiomodulation: Case Series Report,” Photomedicine and Laser Surgery 35, no. 8 (2017): 432–441. 28186867 10.1089/pho.2016.4227 PMC5568598

[101] F. Salehpour , S. Gholipour‐Khalili , F. Farajdokht , et al., “Therapeutic Potential of Intranasal Photobiomodulation Therapy for Neurological and Neuropsychiatric Disorders: A Narrative Review,” Reviews in the Neurosciences 31, no. 3 (2020): 269–286. 31812948 10.1515/revneuro-2019-0063 PMC7138738

[102] J. Song , H. Lee , E. G. Jeong , K. C. Choi , and S. Yoo , “Organic Light‐Emitting Diodes: Pushing Toward the Limits and Beyond,” Advanced Materials 32, no. 35 (2020): e1907539. 32142190 10.1002/adma.201907539

[103] D. Li , S. Liu , T. Yu , et al., “Photostimulation of Brain Lymphatics in Male Newborn and Adult Rodents for Therapy of Intraventricular Hemorrhage,” Nature Communications 14, no. 1 (2023): 6104. 10.1038/s41467-023-41710-y PMC10541888 37775549

